# Galectin-3 immunodetection in follicular thyroid neoplasms: a prospective study on fine-needle aspiration samples

**DOI:** 10.1038/sj.bjc.6602822

**Published:** 2005-10-25

**Authors:** J F Collet, I Hurbain, C Prengel, O Utzmann, F Scetbon, J F Bernaudin, A Fajac

**Affiliations:** 1 Laboratoire d'Histologie-Biologie Tumorale, hôpital Tenon, UPRES EA 3499, Université Pierre et Marie Curie, Paris, France; 2 Cabinet de Cytopathologie, Paris, France; 3 C.C.R, Créteil, Paris, France; 4 CIM Duroc, Paris, France

**Keywords:** galectin-3, thyroid, follicular neoplasms, cytology, fine-needle aspiration

## Abstract

Fine-needle aspiration cytology, which is well established to be accurate for the diagnosis of thyroid cancer, may be inconclusive for the follicular thyroid neoplasms. As galectin-3 was suggested to be a marker of malignant thyrocytes, we investigated whether this protein might be helpful in the diagnosis of aspirates classified as undeterminate by cytology. After establishing an easy processing of aspirates for galectin-3 immunodetection, a series of aspirates categorised as benign (*n*=63), malignant (*n*=17) or undeterminate (*n*=34) was prospectively analysed for galectin-3. Only the patients with malignant or undeterminate lesions underwent surgery. Most lesions (86%) diagnosed as malignant by cytology or after surgery were positive for galectin-3. The majority of lesions (94%) classified as benign by cytology or after surgery was negative for galectin-3. The positive and negative predictive values were 83 and 95%, respectively. When focusing on the undeterminate lesions, the sensitivity and specificity were 75 and 90%, respectively, while the positive and negative predictive values were 82 and 87%, respectively. The specificity and the positive predictive value were higher (100%) when considering the percentage of stained cells. Altogether these results show that galectin-3 constitutes a useful marker in the diagnosis of thyroid lesions classified as undeterminate by conventional cytology.

Thyroid nodules are very frequent in the population and their diagnosis by fine-needle aspiration cytology has been well established to be highly accurate to discriminate malignant from benign lesions (Dwarakanathan *et al*, 1989; Baloch *et al*, 1998). However, among the various thyroid lesions, the so-called follicular neoplasms, which include follicular adenomas and follicular carcinomas, may constitute a diagnostic problem, as cytological findings may be not sufficient to assess malignancy. In such cases, many patients are referred to surgery to perform a histological diagnosis of thyroid nodules and to look for invasion through the tumour capsule or the blood vessels. Given that only about 20% of such undeterminate thyroid lesions will be malignant, a rather high proportion of patients could have been dispensed from surgery. There is therefore a need for better identifying thyroid lesions classified as undeterminate by routine cytology.

Several molecular markers have been investigated in order to improve the diagnosis of follicular thyroid lesions such as integrin, E-cadherin, HBME1 (Serini *et al*, 1996; Sack *et al*, 1997). Galectin-3, which is a 31 kDa member of the *β*-galactoside-binding proteins, seems to be of special interest. This lectin is both intracellular, where it is located in the nucleus, cytoplasm and on the cell surface and extracellular as it can be secreted into the extracellular matrix. Galectin-3 interacts with intracellular glycoproteins such as *β*-catenin (Shimura *et al*, 2004), cell surface molecules (Inohara *et al*, 1996) and extracellular matrix proteins (Ochieng *et al*, 1999). It is involved in various physiological and pathological processes, among them cell growth (Inohara *et al*, 1998), apoptosis (Akahani *et al*, 1997), tumour progression and metastasis (Danguy *et al*, 2002; Takenaka *et al*, 2004).

One recent study showed for the first time on a large series of thyroid nodules that galectin-3 as assessed by immunodetection appeared to be a reliable marker of well-differentiated thyroid carcinoma with high sensitivity and specificity (Bartolazzi *et al*, 2001). However, very few data exist focusing on fine-needle aspiration thyroid samples classified as undeterminate by conventional cytology and the potential help of galectin-3 in better identifying this subset of lesions. In addition, there are some technical problems that limit performing routine galectin-3 immunodiagnosis on fine-needle aspiration samples. Indeed, management of the specimen for galectin-3 immunodiagnosis needs to be no time-delayed and galectin-3 immunodetection has been reported most often for cell blocks, that is, paraffin-embedded cytological samples (Bartolazzi *et al*, 2001; Saggiorato *et al*, 2001) than for fresh smears.

In the present study, the potential interest of galectin-3 in the diagnosis of thyroid nodules categorised as undeterminate by cytology was evaluated in a prospective series of fine-needle aspirates. The optimal processing of fine-needle aspiration thyroid samples for subsequent galectin-3 immunodetection was first defined in order to be realised in routine medical practice.

## MATERIALS AND METHODS

### Specimens

Fine-needle aspiration thyroid nodules obtained from 2002 to 2005 were prospectively analysed for galectin-3. Aspiration was realised with a 26 or 27 gauge needle either under ultrasound control or not when the nodule was palpable. The obtained material was spread on slides. For each sample, a sufficient number of slides was stained with May Grünwald Giemsa (MGG) for routine cytological diagnosis, while at least two smears were realised for subsequent galectin-3 immunocytochemistry.

The prospective analysis concerned the three types of cytological samples, that is the benign, the malignant and the samples classified as undeterminate. The term undeterminate meant that no definitive cytological diagnosis was possible. During the period of investigation, more than 1500 fine-needle aspirations of thyroid nodules were performed. As expected, most of these samples were undoubtedly benign at cytological diagnosis. After having observed that galectin-3 was always negative in our first series of benign cases, we then did not systematically analysed all the benign lesions for galectin-3 but focused on lesions which although unambiguously benign were highly cellular.

The samples subsequently analysed for galectin-3 (*n*=135) consisted of 63 benign, 17 malignant and 55 undeterminate lesions. In four cases of malignant lesions, the analysed material was lymph nodes instead of thyroid tissue as no thyroid nodule was seen by echography. The cytological diagnosis of these lesions was unambiguously papillary thyroid carcinoma.

Indication for surgery was based only on clinical and MGG cytological findings without taking into account the results of galectin-3 immunodetection. Consequently, only the patients with lesions diagnosed as malignant or undeterminate by cytology were supposed to be referred to surgery. Among the 55 patients whose lesions were classified as undeterminate, 34 underwent surgery because of the clinical context. The remaining 21 patients were carefully followed-up or were not operated on by the time we sent this manuscript. Therefore, analysis of galectin-3 results was performed for the lesions diagnosed as benign or malignant by cytology and the subset of undeterminate lesions for which histological diagnosis was available (*n*=114).

The cytological diagnosis of benign lesions was ‘benign follicular lesions’ based on the presence of unambiguously benign cytological findings such as large monolayered sheets of cohesive follicular epithelial cells and abundant colloid. When the slightest atypia were found, the samples were diagnosed as undeterminate. No thyroiditis was included in the present study. The total number of patients who underwent surgery was 51, including 34 lesions diagnosed as undeterminate and 17 lesions diagnosed as malignant by cytology. The histological diagnosis of these lesions is summarised in Table 1.

### Cell culture

The papillary thyroid carcinoma BCPAP cell line (Fabien *et al*, 1994) was grown in DMEM supplemented with 10% heat-inactivated foetal calf serum, 100 IU ml^−1^ penicillin, 25 *μ*g ml^−1^ streptomycin and 2 mM glutamine. The culture was maintained at 37°C in a humidified atmosphere of 5% CO_2_ and 95% air.

### Processing of the aspirates

We determined the optimal processing of fine-needle aspiration samples for subsequent galectin-3 immunodetection. Specimens were first tested for transport in CytoLyt (Cytyc, MA, USA). Samples were aspirated, put in CytoLyt then centrifuged on slide and tested for galectin-3. Samples were also spread on slides and analysed for galectin-3 after air-drying only.

To define the optimal time between aspiration of samples and galectin-3 detection, we tested the human papillary thyroid carcinoma BCPAP cells. These cells were incubated in CytoLyt for different times, then centrifuged on slides and tested for galectin-3. They were also centrifuged on slides without prior incubation in CytoLyt, dried on air for different times and then tested for galectin-3.

### Galectin-3 immunocytochemistry

Cells on slides were fixed in acetone at 4°C for 10 min followed by 10% formalin at room temperature for 10 min. After blockage of nonspecific binding sites with 30% normal human AB serum, cells were incubated overnight at 4°C with anti-human galectin-3 NCL-GAL3 mouse monoclonal antibody (Novocastra, UK) diluted 1 : 100. Cells were then incubated with EnVision-peroxydase detection kit as recommended by the manufacturer (Dako, France). Enzymatic activity was visualised with 3,3′-diaminobenzidine (Dako). Slides were then washed, counterstained with haematoxylin and mounted in aqueous medium. For each experiment, the human papillary thyroid carcinoma BCPAP cell line was used as positive control. For each sample, a negative control was realised, which consisted of substitution of the primary antibody by an isotype-matched mouse nonimmune IgG1. Positivity for galectin-3 was assessed by two independent investigators taking into account the percentage of cells exhibiting staining in the cytoplasm and/or at the plasma membrane. The positive cases were classified+when staining was observed in less than 20% cells, ++ for staining in 20–50% cells and +++ for staining in more than 50% cells.

### Statistics

Sensitivity, specificity, positive and negative predictive values of galectin-3 immunodiagnosis were calculated as follows. Sensitivity was defined as the ratio of the number of carcinomas that were galectin-3 positive to the total number of carcinomas. Specificity was defined as the ratio of the number of benign lesions, which were galectin-3 negative to the total number of benign lesions. The positive predictive value was calculated as the ratio of the number of galectin-3 positive carcinomas to the total number of galectin-3 positive lesions. The negative predictive value was calculated as the ratio of the number of galectin-3 negative benign lesions to the total number of galectin-3 negative lesions.

## RESULTS

### Optimal processing of the aspirates before galectin-3 immunocytochemistry

**Figure 1:**
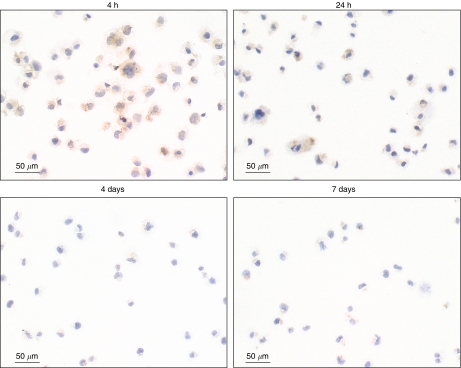
Galectin-3 immunodetection in CytoLyt-preserved BCPAP cells. The human papillary thyroid carcinoma BCPAP cells were analysed for galectin-3 after incubation in CytoLyt for different times.

**Figure 2:**
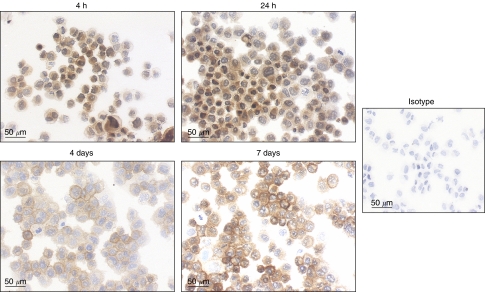
Galectin-3 immunodetection in air-dried BCPAP cells. BCPAP cells were analysed for galectin-3 after air-drying on slides for different times.

We found that detection of galectin-3 was possible for aspirated samples put in CytoLyt. This transport medium allowed detection of the protein for samples that have been in CytoLyt as long as 48 h. To examine whether the delay between fine-needle aspiration and galectin-3 immunodetection could be longer than 48 h, we tested the human papillary thyroid carcinoma BCPAP cell line for galectin-3 after incubation in CytoLyt for different times (4 h–7 days). It was clearly shown that galectin-3 labelling decreased with time to be almost absent after 7 days (Figure 1). Consequently, we then tested a new protocol where BCPAP cells were not transported in a medium but only air-dried on slides. We found that galectin-3 labelling was clearly visible for each time tested and even as long as 7 days (Figure 2). Therefore, the fine-needle aspiration samples subsequently analysed were only air-dried smears, which were tested for galectin-3 within 7 days after aspiration.

### Galectin-3 immunodetection in all aspirates

**Figure 3:**
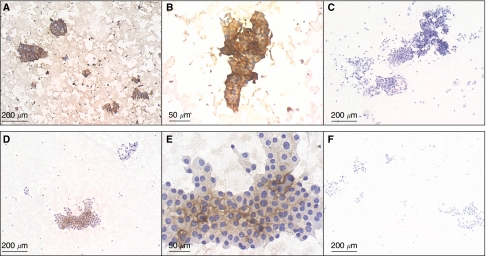
Galectin-3 immunodetection in air-dried fine-needle aspiration thyroid samples. Examples of a papillary carcinoma (**A**–**C**) and an undeterminate follicular lesion exhibiting oncocytic metaplasia (**D**–**F**), which were positive for galectin-3. Samples were labelled with NCL-GAL 3 mouse monoclonal antibody (**A**, **B**, **D**, **E**) or an isotype-matched mouse IgG1 (**C**, **F**). (**B** and **E**) Higher magnification of (**A**) and (**D**), respectively.

A series of 114 fine-needle aspirates processed as reported above and categorised as benign (*n*=63), malignant (*n*=17) or undeterminate (*n*=34) was prospectively analysed for galectin-3. Typical examples of galectin-3 labelling using NCL-GAL3 mouse monoclonal antibody are shown in Figure 3. Detection of galectin-3 was observed in 30 cases among which three were classified as benign, 16 as malignant and 11 as undeterminate by cytology. The percentage of positive cells was variable from 20 to 100%. Galectin-3 staining was always observed in the cytoplasm. In half cases (*n*=15), cytoplasmic staining was associated with plasma membrane staining. No nuclear staining was seen.

As it was a prospective study, the patients with lesions classified as benign due to the presence of undoubtedly benign findings such as large monolayered sheets of cohesive epithelial cells and abundant colloid were not operated on. Histological diagnosis was available only for lesions diagnosed as undeterminate or malignant by cytology (Table 1). As expected, the histological diagnosis of lesions classified as malignant by cytology was malignancy. Histology of undeterminate lesions consisted of 22 benign and 12 malignant lesions. Among the 11 indeterminate lesions positive for galectin-3, nine were diagnosed as malignant and two as benign by histology. Galectin-3 results according to histological diagnosis are summarised in Table 2.

Considering all the malignant lesions (*n*=29), most lesions (*n*=25, 86%) were positive for galectin-3 (Table 2). Positive cases were papillary carcinomas (including follicular variants) and one trabecular carcinoma. In most samples, the staining was observed in a high percentage of cells, in more than 50% cells in all cases except two and more than 80% cells in 16 cases. The negative cases consisted of one follicular variant of papillary carcinoma, two follicular carcinomas and one medullary carcinoma.

Taking into account all the lesions classified as benign either by cytology or after surgery (*n*=85), the majority of benign lesions (94%) was negative for galectin-3. Concerning the five positive lesions, the percentage of labelled cells was less than 50% in all samples except one.

The sensitivity, specificity, positive and negative predictive values for galectin-3 in all the aspirates were 86, 94, 83, 95%. When considering samples to be positive when more than 50% cells were positive for galectin-3, the specificity as well as the positive predictive value were higher (99 and 96%, respectively) while the negative predictive value was similar (93%); however, the sensitivity was lower (79%).

### Galectin-3 immunodetection in undeterminate aspirates

When focusing the analysis of galectin-3 results on undeterminate lesions, galectin-3 was detected in most malignant lesions and absent in the majority of benign lesions (Table 3). The sensitivity, specificity, positive and negative predictive values for galectin-3 calculated for this subset of aspirates are reported in Table 4. If samples were considered to be positive when labelling was observed in more than 50% cells, again the specificity and the positive predictive value were higher (Table 4). The two undeterminate galectin-3 positive samples, which were diagnosed as benign, that is one follicular adenoma and one oncocytic adenoma, exhibited positivity in 30 and 20% cells, respectively.

## DISCUSSION

Since the first large series of Bartolazzi *et al* (2001), several studies have dealt with the potential usefulness of galectin-3 to better evaluate thyroid lesions. There has been some controversial results with studies showing galectin-3 to be a very interesting marker of malignancy (Orlandi *et al*, 1998; Saggiorato *et al*, 2001; Nascimento *et al*, 2001; Oestreicher-Kedem *et al*, 2004), including molecular profiling studies of follicular thyroid lesions (Finley *et al*, 2004) and others reporting that galectin-3 cannot discriminate benign from malignant thyroid lesions (Mehrotra *et al*, 2004; Mills *et al*, 2005). The reasons for this discrepancy might be related to differences in technical procedures, in the type of thyroid lesions analysed and to insufficient number of samples tested. In a very recent review on the optimal management of thyroid nodules (Castro and Gharib, 2005), galectin-3 was mentioned as an immunohistochemical marker having shown to be potentially interesting in preliminary studies. Therefore, there is a need for other investigations dealing with galectin-3 in thyroid lesions.

Most of the published studies concern retrospective analyses made on histological specimens or paraffin-embedded cell blocks. They do not evaluate the interest of galectin-3 as a marker of malignancy in the subset of lesions for which no diagnosis is possible by cytology. The present work was a prospective study and focused on samples classified as underterminate by cytology, which are the samples of concern.

First of all, we optimised a galectin-3 immunocytochemical method for fine-needle aspiration thyroid samples. We show here that galectin-3 immunodetection is possible in routine medical practice especially as an office procedure. The processing of the specimens is easy as only air-dried smears are required. This procedure is shorter and less expensive than imunohistochemistry performed on paraffin-embedded cell blocks, which is the technique most often reported for fine-needle aspiration samples (Bartolazzi *et al*, 2001; Saggiorato *et al*, 2001). The delay between aspiration and galectin-3 immunocytochemistry can be as long as 7 days.

It is important to note that we used a biotin-free detection system. Indeed, certain thyroid cells, especially oncocytic cells that are frequently present in follicular adenomas, are known to be rich in biotin and could be responsible for false-positive results in biotin-based detection systems (Herrmann *et al*, 2002).

We always observed galectin-3 labelling in the cytoplasm and never in the nucleus of follicular cells. Cytoplasmic staining has been reported to be associated with nuclear staining in some cases mainly papillary carcinoma (Orlandi *et al*, 1998; Saggiorato *et al*, 2001). However, most authors agree that the most significant localisation of galectin-3 in thyroid lesions is cytoplasm as nuclear staining can be observed in a high number of benign lesions namely follicular adenoma (Orlandi *et al*, 1998; Saggiorato *et al*, 2001). In addition, various studies although performed on other tumours than thyroid tumours, such as prostate and colon carcinoma, suggest that galectin-3 plays a dual role according to its localisation: nuclear galectin-3 would play antitumour functions while cytoplasmic galectin-3 would promote tumour progression (Lotz *et al*, 1993; Honjo *et al*, 2000; Van den Brule *et al*, 2000; Califice *et al*, 2004).

We also observed staining of galectin-3 at the plasma membrane in half cases. The localisation of galectin-3 (previously called Mac-2) at the cell surface has been previously reported (Sato and Hughes, 1994; Inohara *et al*, 1996). The finding of plasma membrane staining is in accordance with the role of galectin-3 in cell–cell and cell–matrix adhesion. For example, galectin-3 is known to be involved in cell–cell interactions by binding to Mac-2 binding protein (Inohara *et al*, 1996) and in cell–matrix interactions by binding to elastin (Ochieng *et al*, 1999).

Without considering the percentage of stained cells in samples, calculation of sensitivity, specificity, positive and negative predictive values for galectin-3 for all the aspirates yielded high values (>90%) for the specificity and the negative predictive value. When focusing on undeterminate lesions, the specificity and the negative predictive value were also the highest values and were almost equal to 90% (Table 4). The fact that these values were lower for this subset of lesions is probably due to the relatively low number of cases. However, of interest was the result of the relatively high negative predictive value of galectin-3 in this subset of lesions (87%). Indeed, this indicates that the absence of galectin-3 labelling was associated with the presence of benign lesions in most cases.

The absence of galectin-3 detection in thyroid carcinomas and the presence of galectin-3 in benign thyroid lesions have been previously reported by others (Kovacs *et al*, 2003), even in the large study by Bartolazzi, which demonstrated high sensitivity and specificity (>90%) of galectin-3 as a marker of malignant thyroid lesions (Bartolazzi *et al*, 2001). In the present study, a relatively high number of benign lesions were positive for galectin-3, namely in the subgroup of undeterminate lesions, two out of 22 benign lesions (9%). In studies dealing with galectin-3 in thyroid lesions including the largest study of Bartolazzi, positivity for a proportion of benign lesions has always been reported in cytological as well as histological samples. The frequency of positivity is around 10% varying from 4 to 11% (Orlandi *et al*, 1998; Bartolazzi *et al*, 2001; Saggiorato *et al*, 2001). It has been suggested that some of these positive cases might constitute cases undergoing malignant transformation as features suspicious of malignancy such as hypercellularity, increased nucleus–cytoplasm ratio and mitoses were observed although no invasion of the capsule or blood vessels was present (Saggiorato *et al*, 2001; Coli *et al*, 2002).

An interesting finding of the present study is that when galectin-3 labelling was present in benign cases, the percentage of galectin-3 positive cells was lower than in malignant cases. When considering samples to be positive if they contained more than 50% stained cells, the specificity and the positive predictive value were higher than without considering the percentage of stained cells (Table 4). No major modification was found for the sensitivity and the negative predictive value. These results were found when taking into account all the aspirates as well as when focusing on undeterminate samples. These findings suggest that galectin-3 constitutes a better marker of thyroid malignancy if semiquantitative rather than qualitative results are taking into account. We propose that the cutoff value to consider a sample positive or negative for galectin-3 could be 50% of stained cells.

Considering the malignant cases negative for galectin-3, other potentially interesting markers such as HBME1 or cytokeratin 19 (Sack *et al*, 1997; Prasad *et al*, 2005) should be evaluated. The use of two or three markers might be more relevant than just one as recently shown on a series of 37 fine-needle aspiration biopsies using a panel of markers consisting of galectin-3, HBME1 and RET, where none of the 20 malignant tumours was negative for this panel (Rossi *et al*, 2005).

In conclusion, galectin-3 immunodetection in fine-needle aspiration thyroid samples is easy and possible in routine medical practice. This marker is useful in the diagnosis of thyroid lesions classified as undeterminate by conventional cytology, especially if semiquantitative results, that is to say the percentage of positive cells, are taking into account. Galectin-3 allows one to identify the subset of undeterminate lesions with poor risk of malignancy and consequently the subgroup of patients who, if in accordance with the clinics, may be carefully followed-up instead of being referred to surgery.

## Figures and Tables

**Table 1:** Histological diagnosis of lesions classified as undeterminate or malignant by cytology

**Cytology**	**Histology**				
	**Benign (*n*=22)**		**Malignant (*n*=29)**		
	**Follicular adenoma (*n*=14)**	**Oncocytic adenoma (*n*=8)**	**Papillary carcinoma and follicular variants (*n*=25)**	**Follicular carcinoma (*n*=3)**	**Medullary carcinoma (*n*=1)**
*Undeterminate (n*=*34*)					
Follicular lesion (*n*=26)	14		9	3	
Oncocytic lesion (*n*=8)		8			
					
*Malignant (n*=*17*)					
Papillary carcinoma (*n*=16)			16		
Follicular carcinoma (*n*=1)					1

**Table 2:** Galectin-3 results according to histological diagnosis of lesions classified as undeterminate or malignant by cytology

	**Benign (*n*=22)**		**Malignant (*n*=29)**		
	**Follicular adenoma (*n*=14)**	**Oncocytic adenoma (*n*=8)**	**Papillary carcinoma and follicular variants (*n*=25)**	**Follicular carcinoma (*n*=3)**	**Medullary carcinoma (*n*=1)**
Gal 3-positive	1	1	24	1	0

**Table 3:** Galectin-3 results for undeterminate lesions according to their subsequent categorisation as benign or malignant after surgery

	**Benign (*n*=22)**	**Malignant (*n*=12)**
Gal 3-positive	2	9
Gal 3-negative	20	3
		
Gal3 staining in ⩾50% cells	0	8
Gal3 staining in <50% cells	22	4

**Table 4:** Discrimination between benign and malignant lesions by galectin-3 for the aspirates diagnosed as undeterminate by cytology

**Variable**	**Galectin-3**	**Galectin-3 (positivity if ⩾50% stained cells)**
Sensitivity (%)	75	67
Specificity (%)	91	100
Positive predictive value (%)	82	100
Negative predictive value (%)	87	85
